# Unusual Malignant Transformation of Recurrent Sebaceoma. A Case Report

**DOI:** 10.4137/cmo.s477

**Published:** 2008-04-15

**Authors:** Heba Al-Khashnam, Hisham Burezq, Ibrahim Al-Aradi, Humoud Al-Sabah, Khalid Al-Abdulhadi

**Affiliations:** 1 Resident, Al-Babtain Center for Burns and Plastic Surgery, Ibn Sina Hospital, Sabah Health Area, State of Kuwait; 2 Consultant, Plastic and Reconstructive Surgeon, Al-Babtain Center for Burns and Plastic Surgery, Ibn Sina Hospital, Sabah Health Area, State of Kuwait; 3 Consultant, Surgical Dermatologist, Asaad Al-Hamad Dermatology Center, Sabah Health Area, State of Kuwait; 4 Histopathologist, Asaad Al-Hamad Dermatology Center, Sabah Health Area, State of Kuwait; 5 Chairman, Department of ENT and Head and Neck Surgery, Sabah Hospital for Head and Neck and ENT Surgery, Sabah Health Area, State of Kuwait

**Keywords:** sebaceoma, recurrence, malignancy

## Abstract

Sebaceoma is a benign tumor composed of incompletely differentiated sebaceous cells of varying degrees of maturity. Sebaceomas was never reported as a known premalignant lesion.

This is a report of a sixteen year old boy who presented with a malignant transformation of a recurrent sebaceoma which was excised twice by Moh’s surgery. Excision was done with a free margin of 1 cm down to the parotid fascia. Reconstruction was performed on the same set by using cervicofascial flap extending down to the supra-clavicular area. The patient had an uneventful postoperative period apart from distal marginal necrosis of the flap, which healed nicely with conservative measures and daily dressing and was sent to our cancer centre to start his adjuvant radiotherapy.

Previous literature stated that sebaceoma is a distinctive benign tumor. We have presented a case of an unusual malignant transformation of a preauricular recurrent sebaceoma. This indicates that sebaceoma does have a potential risk of malignant transformation. We believe that managing recurrent sebaceoma more aggressively with wide local excision and postoperative adjuvant radiotherapy would provide better prognosis.

## Introduction

Sebaceoma is a benign tumor composed of incompletely differentiated sebaceous cells of varying degrees of maturity. Sebaceoma was never reported as a known premalignant lesion. This is a report of a sixteen year old male patient who presented with a malignant transformation of a recurrent sebaceoma of the right pre-auricular area.

## Case Report

**Figure 1:**
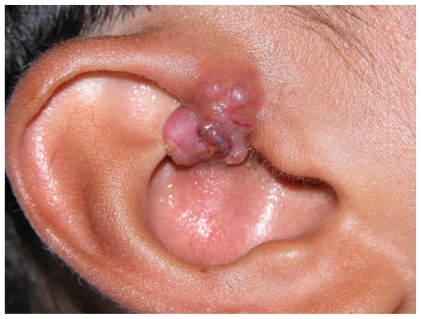
Original leasion.

**Figure 2:**
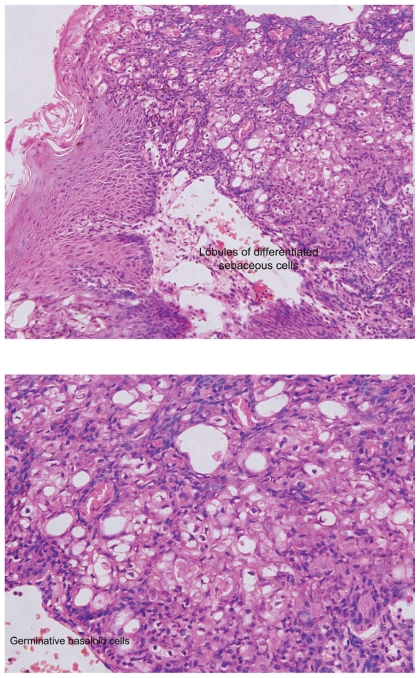
**(A, B). A**) Low power, **B**) High power Slide showed masses of basaloid cells. Some of the masses are attached to the undersurface of the epithelium. These masses show sebaceous differentiation. The lobules of sebaceous glands show Irregular growth pattern. Germinative epitheloid cells predominate and haphazardly arranged. There are multiple keratinous microcysts and few mitotic figures.

A previously healthy sixteen year old male patient presented with a right pre-auricular lesion. This was excised by general surgery department and was reported as a sebaceous cyst. One year later he presented with a friable nodule measuring 1 cm × 0.75 cm in the scaphoid fossa of the right ear and incisional biopsy showed histological features of sebaceoma (Figs. 1, 2). Moh’s surgery was performed twice. The pre-auricular area, superior and inferior crura, triangular fossa and the choncha of the right ear were found to be involved. The resulted defect was reconstructed with a full thickness skin graft.

**Figure 3:**
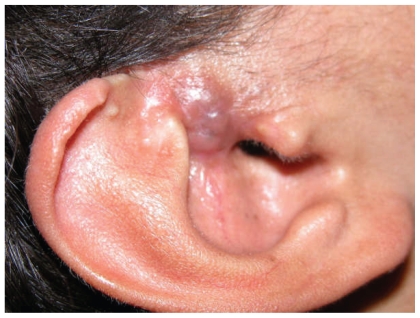
Recurrence of lesion after Moh’s surgery.

**Figure 4:**
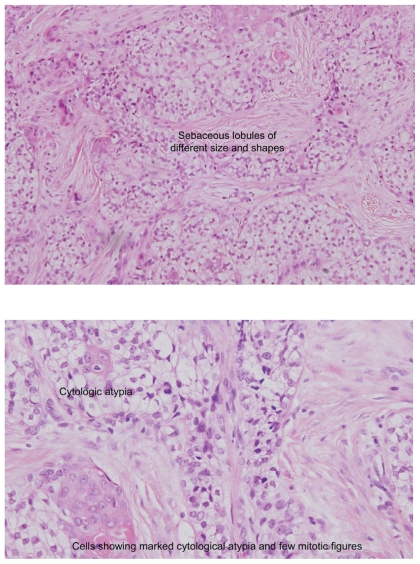
**(A, B). A**) Low power, **B**) High power The biopsy specimen showed marked proliferation of irregular sebaceous lobules of different sizes and shapes in the dermis. The cells of the lobules are mostly undifferentiated and demonstrated marked cylologic atypia with considerable nuclear pleomorphism and few mitotic activities. Some of the sebaceous lobules showed areas composed of atypical keratinizing cells. The overlying epidermis is normal with marked dermal fibrosis. Immunoperoxidase staining showed positive reaction for cytokeratin (CK), focal positive for epithelial membrane antigen (EMA) and negative reaction for carcino-embryonic antigen (CEA).

**Figure 5:**
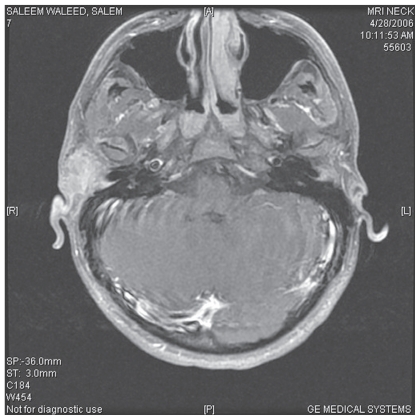
MRI study showed a 3.25 × 2.25 × 4.5 cm mass extending to the Masseter muscle and petrous bone medially, the cartilaginous part of the external auditory meatus posteriorly and the upper border of the right parotid gland inferiorly.

One year later the patient presented with a 0.5 cm × 0.75 cm soft purplish lesion. Incisional biopsy was consistent with sebaceous carcinoma (Figs. 3, 4). MRI study showed a 3.25 × 2.25 × 4.5 cm mass extending to the Masseter muscle and petrous bone medially, the cartilaginous part of the external auditory meatus posteriorly and the upper border of the right parotid gland inferiorly. One prominent ipsilateral lower cervical lymph node was felt, but fine needle examination showed no malignant metastasis (Fig. 5).

**Figure 6:**
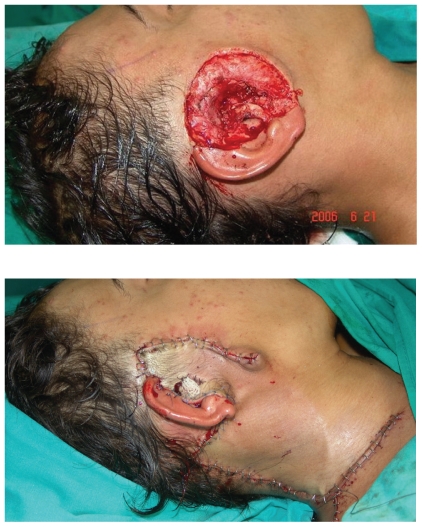
**(A, B).** A) Large defect created after wide local excision, B) reconstructed by a cervico-fascial flap extending down to the supra-clavicular area.

This was managed by wide local excision with postoperative radiotherapy. Excision was done with a free margin of 1 cm down to the parotid fascia. Local cervicofascial rotational flap area (Fig. 6) was used to reconstruct the resulted defect. Pathological analysis revealed T4 N0 M0 sebaceous carcinoma of the pre-auricular area with microscopic infiltration of the parotid fascia. All other margins were free. The cervical lymph node showed reactive changes and benign salivary gland inclusions without evidence of tumor metastasis. Further excision of the superficial part of the parotid was avoided to preserve the main trunk of the facial nerve and its branches.

The patient had uneventful postoperative period apart from distal marginal necrosis of the flap, which healed nicely with conservetive measures and daily dressing and was sent to our cancer centre to start his adjuvant radiotherapy. The patient was followed for 13 months with no evidence of recurrence.

## Discussion

Sebaceoma is a new term proposed to designate a distinctive rare benign neoplasm of adnexal epithelium with differentiation toward sebaceous cells.^1^ Most authors^1^–^4^ described this lesion as solitary or sometimes multiple nodules or plaques that have the appearance of a basal cell epithelioma and may show ulceration with a rolled, pearly border. Occasional tumours are more deeply located and appear cystic. The colour may be fleshy or of a waxy, yellowish hue, and the surface may be verrucose. Most lesions are located in the head and neck area. It usually grows slowly, but a sudden increase in growth rate can occur. The best treatment of sebaceoma is surgical excision.

The histologic spectrum extends from that seen in sebaceous adenoma to lesions that may be difficult to distinguish from sebaceous carcinoma.^4^ Basal cell carcinoma with sebaceous differentiation is an uncommon event; most such cases also represent sebaceoma.^6^

Sebaceous carcinoma is a rare, aggressive, malignant tumor that is derived from adnexal epithelium of sebaceous glands^76^ and complete surgical excision is required.^8^ Reports of excellent results with Moh's surgery suggest that this maybe the treatment of choice.^9^

In a clinicopathological study of five cases with sebaceous carcinoma arising in nevus sebaceous of Jadassohn, it has been shown that sebaceous carcinoma was always accompanied by other benign or malignant adnexal lesions such as sebaceoma, syrigocystadenoma papilliferum, syringocystadenocarcinoma papilliferum, trichoblastoma, trichlemmoma, desmoplastic tricholemmoma, or syrigoma.^10^ The coexistance of sebaceous carcinoma, trichoblastoma, and sebaceoma was also reported in a nevus sebaceous.^11^ In our case report Moh’s surgery was performed in the histopathological diagnosis of sebaceoma, and a coexisting sebaceous carcinoma will be hard to miss. However, the possibility of the presence of sebaceoma and sebaceous carcinoma concurrently cannot be dismissed.

## Conclusion

We have presented a case of an unusual malignant transformation of a preauricular recurrent sebaceoma. This indicates that sebaceoma could have a potential risk of malignant transformation. Managing recurrent sebaceoma more aggressively with wide local excision and postoperative adjuvant radiotherapy would possibly provide better prognosis.
